# Testing the applicability of tagging the Great crested newt (*Triturus cristatus*) using passive integrated transponders

**DOI:** 10.1371/journal.pone.0219069

**Published:** 2019-07-08

**Authors:** Lukáš Weber, Marek Šmejkal, Daniel Bartoň, Martin Rulík

**Affiliations:** 1 Department of Ecology and Environmental Sciences, Faculty of Science, Palacký University, Olomouc, Czech Republic; 2 Biology Centre of the Czech Academy of Sciences, Institute of Hydrobiology, České Budějovice, Czech Republic; Universitat Zurich, SWITZERLAND

## Abstract

Tracking individual animals with small-sized passive integrated transponder tags (PIT tags) has become a popular and widespread method, one which can be used for investigating life history traits, including dispersal patterns of small protected animals such as newts. In this study, we tested the applicability of PIT tag usage for individual marking with the Great crested newt (*Triturus cristatus*) as a model amphibian species, and to test the detection of the newts in nature using a passive telemetry system. Clove oil was used as an anaesthetic before surgery. We implanted PIT tags under the skin of 140 newts. The survival rate of newts was 98.57%. X-ray images were taken to check the exact positions of the PIT tags. Since approximately 15.71% of the newts were capable of expelling the tag from their bodies, tag loss has to be accounted for in future behavioural studies dealing with newts and other amphibians potentially capable of frequent tag expulsion. Lastly, we detected by passive telemetry 97 individuals out of 100 released into a natural breeding pond. Males had higher activity (13 detected males vs 7 females per hour) than females, thus males could be detected if present with more certainty. The result of the movement behaviour showed that e.g. the male of *T*. *cristatus* in a breeding pond can travel up to 20 m in 78 seconds. In summary, this promising method could allow the automatic data collection of marked newts in aquatic as well as in terrestrial biotopes, providing data on their dispersal, diurnal activity and movement behaviour.

## Introduction

Amphibian populations have been in fact declining for several decades [1]. In our study we took the Great Crested Newt (*Triturus cristatus*), as a model amphibian species, which shows a significant decline in populations [2, 3, 4]. One of the critical components of conservation biology is movement behaviour, affecting how individuals interact spatially with other organisms and their environment [5, 6]. Animals may move to avoid predators, acquire resources, find mates, or to escape high conspecific density [7]. Movement studies are unique in their capacity to investigate processes at a variety of levels, including individual, population, and community [8]. Movement behaviour such as dispersal, foraging, and migration is poorly understood in many taxa (e.g. amphibian) due to the incompatibility of traditional active tracking methods (colouration patterns, radio transmitters) with long-term observations [9]. Photo-matching is popular and largely used method for individual identification in amphibian species [10, 11]. However, this method can be time-consuming, even using automatic processes for photographic re-identification. It is necessary to recapture the animal and handle it again [12, 13]. Recent developments in radio-frequency identification (RFID) and passive integrated transponder (PIT) tags have introduced a method suitable for documenting dispersal patterns and also quantifying habitat use of many small-bodied vertebrates, including amphibians and fishes [11, 14, 15, 16, 17].

RFID passive telemetry automatically recording PIT tag presence is today an available and increasingly popular option for investigating life histories, including the above-mentioned dispersal patterns of small animals [18, 19]. PIT tags have been widely used in ornithological research [20], including studies providing data of nestling diet [21], possible differences in the incubation behaviour of adults at successful and unsuccessful nests [22], hypotheses (better option, incompatibility or asynchronous arrival) best explaining divorce in the common tern [23], investigation of prospecting behaviour of pied flycatchers (*Ficedula hypoleuca*) at conspecific nests within a short time period following a simulated predator visit [24] and if seasonal date of initial arrival at breeding grounds predicts the individual age at first reproduction of the common tern [25], etc. Since PIT tags’ first usage in biology in the mid-1980s, the numbers of studies using them has continually increased [26]. Passive telemetry could provide amazing and new opportunities to study amphibians, because of their ability to identify an individually marked animal for a long time and could bring us many of information about life history (e.g. diurnal activity and differences in movement between male and female, filopatry, metapopulation distributions, etc.) [27, 28, 29, 30]. In this contribution, we want to apply a method, which newly used subdermally implanted PIT tags in the Great crested newt (*T*. *cristatus*) as is threatened and declining species in the Europe [29]. One of the problems of tagging could be the choice of appropriate anaesthetics. A variety of anaesthetics have been used in amphibian studies with varying effectiveness and duration of anaesthesia, including zolazepam, methoxyflurane, isoflurane, propofol, tricaine methanesulfonate, barbiturates and clove oil [31]. We decided to use clove oil as an anaesthetic. We also want to determine the survival rate of *T*. *cristatus* individuals carrying PIT tags for 48 h in the lab and in the pond. The other aim was to answer whether some PIT tags can be expelled. This could be a problem for future studies aiming to estimate population parameters of given species, potentially leading to overestimation of population size in mark-recapture models. Lastly, we tested tracking of *T*. *cristatus* with passive telemetry in a natural breeding pond; we tested whether males differ from females in their activity and whether the newts are more active during the day or night.

## Methods

The Great crested newts (*T*. *cristatus*) used in this study were obtained in a breeding pond located in Tovéř (49.6405478N, 17.3281758E, altitude 235 m), 7 km northeast of Olomouc in the eastern part of the Czech Republic. This locality hosts breeding populations of both *T*. *cristatus* and *Lissotriton vulgaris*, and its surface area reaches 500 m^2^ during the spring, with a maximum water depth of approximately 1.9 m; sometimes in warm summers it completely dries up. The littoral zone is dominated by pondweed (*Lemna minor*) and submerged grasses are present around the pond. According to the Habitat Suitability Index (HSI) [32] assessing the suitability habitat for GCN occurrence, the Tovéř locality falls into the category “good”. The experiment started at the end of the breeding season; however the pond surprisingly dried up, because of low precipitation and higher temperature that year. In May 2018, we caught 140 individuals of *T*. *cristatus*, 64 females and 76 males, by using 10 standard funnel prism shaped traps commonly used for capturing amphibians [33, 34, 35]. We took photos of the captured *T*. *cristatus* with their characteristic patterns on the belly (for double marking), then they were placed into several separate aquariums (80 l, water temperature 18 °C, submerged vegetation, gravel and sand at the bottom) in an air-conditioned room (20 °C) for 10 days. The daylight/dark pattern was set to 14/10 h according to local natural summer sunrise and sunset. The *T*. *cristatus* individuals were fed by frozen blocks of bloodworms (*Chironomus plumosus*) thawed one hour prior to feeding. *T*. *cristatus* were left to acclimate for 10 days before the experiment started. This study was carried out in strict accordance with the recommendations by the preamble to Act No 246/1992 Czech Law Coll., on the protection of animals against cruelty, the basic law related to animal protection governing the activities of all the state authorities of animal protection in the Czech Republic, such as the Ministry of Agriculture, including the Central Commission for Animal Welfare, and the veterinary administration authorities. The protocol was approved by the Ministry of Agriculture (protocol no. 23/2016). All surgery was performed under clove oil anaesthesia, and all efforts (short time of manipulation in a sterile environment, etc.) were made to minimize suffering. The phenolic compound eugenol, the active component of clove oil, is an effective anaesthetic for amphibians, and can be applied by immersion, potentially making it suitable for work in the field with species that are difficult to handle [36]. After consulting a veterinarian, by protocol (23/2016 –see above), the basic criterion for humane endpoints were impaired ambulation, which prevents animals from reaching food or water, excessive weight loss and extreme emaciation, lack of physical or mental alertness, difficult laboured breathing, or prolonged inability to remain upright [37].

**Fig 1:**
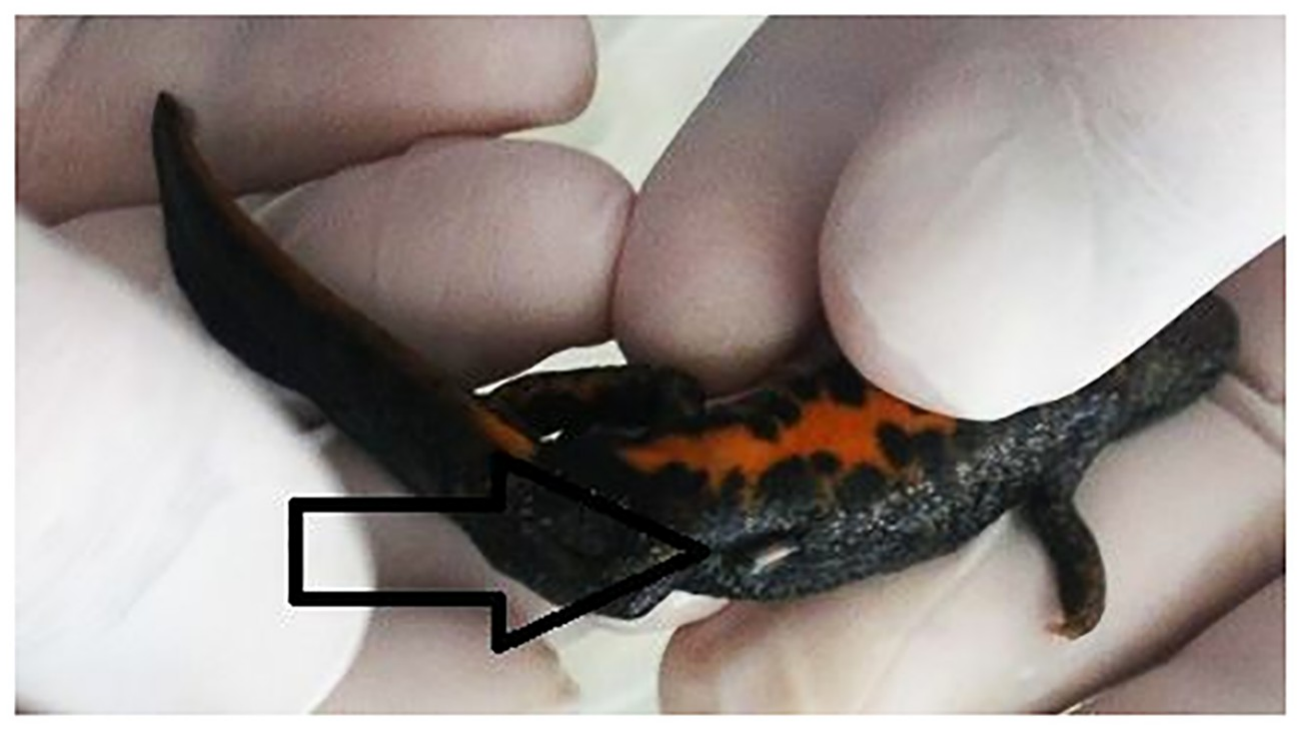
Position of PIT tag. Insertion position of a PIT tag into the body cavity of the *T*. *cristatus*.

**Fig 2:**
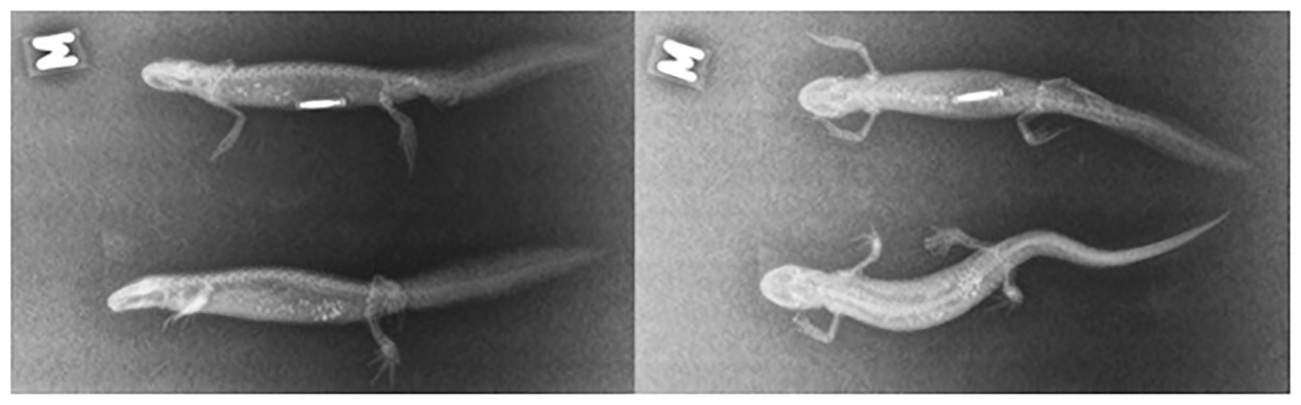
X-ray images. X-ray images of two *T*. *cristatus* in lateral (left picture) and in dorsal (right) position. The upper newt is a male with a PIT tag, the lower newt is a female without PIT tag.

In this study, we implanted 140 PIT tags (12.0 mm x 2.12 mm, 0.1 g half duplex (HDX), Oregon RFID; Portland, Oregon, USA) under the skin of 140 *T*. *cristatus*. Each individual was anaesthetised by a 7–10 minute immersion in a clove oil solution prepared by adding 0.025 ml of 100% oil extract into 400 ml of dechlorinated water at 20 °C, in a small plastic box with cover [38]. Finally, anaesthetic depth was based on our previous experiment with the Alpine bullhead (*Cottus poecilopus*) and Smooth newt (*Lissotriton vulgaris*). The weight of *T*. *cristatus* falls in the range between those two species. Anaesthesia was achieved after 5–10 minutes. In the clove oil solution, *T*. *cristatus* tended to behave normally during the first 5 minutes, without any signs of stress (e.g. accelerated breath), until they started to turn upside-down. We did not observe any prolapse or respiratory depression. After removal from the plastic box, *T*. *cristatus* were gently put on one side and a short (2 mm) lateral subdermal incision with a sterile scalpel was performed in the side of the body, half-way between the belly and the back, approximately 5 mm before the hind legs. A sterile needle with a blunt end was inserted into the incision to create space for the PIT tag, which was gently inserted into the cavity (Fig 1). We did not use a medical adhesive to seal the site of injection because of risk of rupture [11]. The surgical procedure took less than 3 minutes; afterwards the *T*. *cristatus* were laid onto a wet substrate. The anaesthetized individuals of *T*. *cristatus* awoke after 60–90 minutes. After that, *T*. *cristatus* were left under control (48 h) by an internet protocol (IP) camera, and in case of abnormal behaviour, the PIT tag was removed from the body. The presence of the PIT tag was checked individually by an HDX hand reader (Portland, Oregon, USA). One week later, X-ray (79 kV and 0.8m As) images were taken to check the exact positions of the PIT tags (Fig 2). During the X-ray the *T*. *cristatus* were once again anaesthetised using the clove oil solution by the procedure described above.

**Fig 3:**
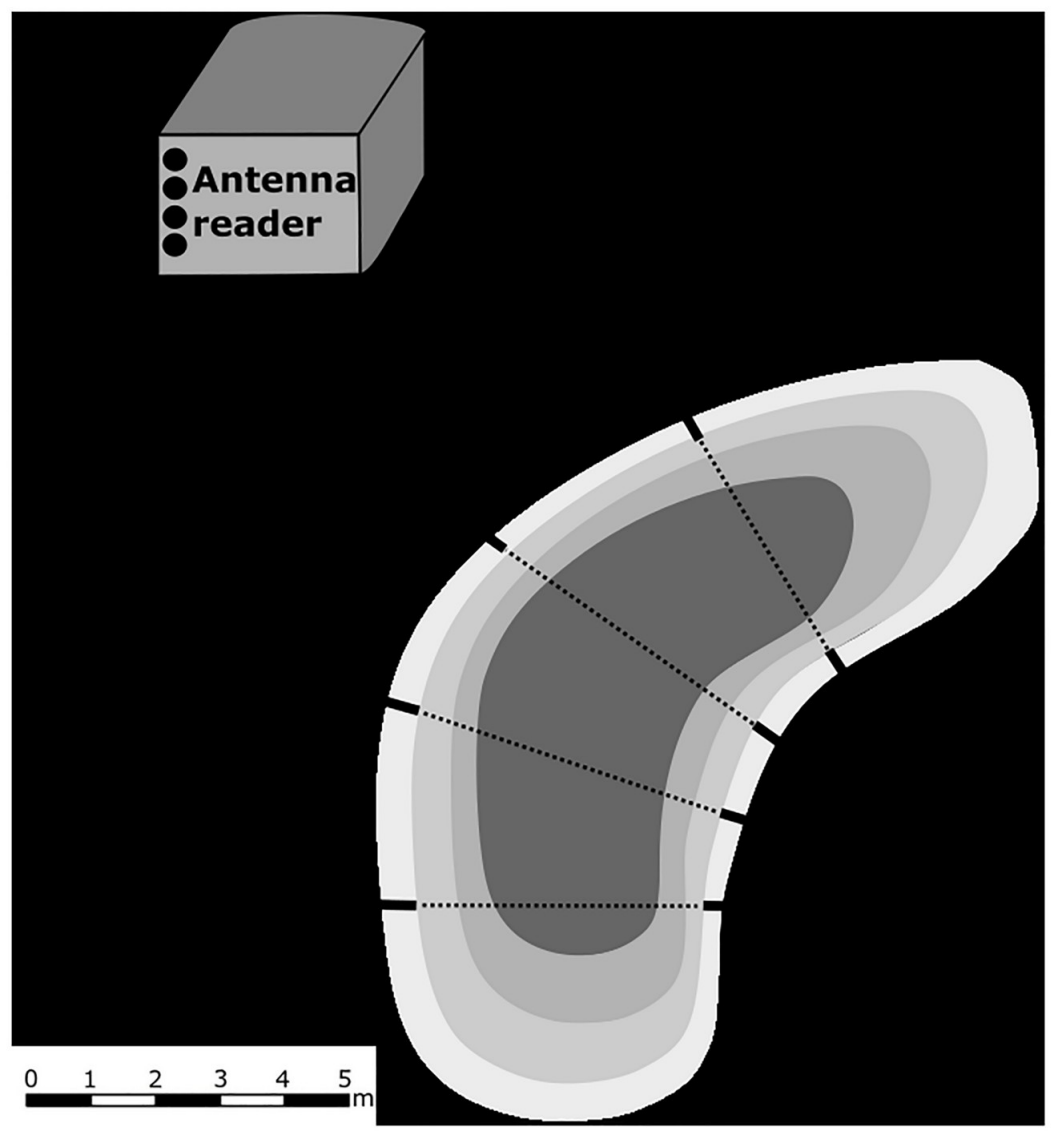
Passive telemetry system. Position of the passive telemetry system with four antennas and reader in the Tovéř breeding pond. In order to guide *T*. *cristatus* through antennas, we used plastic barriers on the edges of the pond.

In order to explore the feasibility of studying *T*. *cristatus* in its aquatic phase using a passive telemetry system, we tracked the presence of 100 randomly selected of 140 PIT tagged individuals at the place where the newts were originally caught via a passive telemetry system (Oregon RFID, Multi-Antenna HDX Reader). Of the remaining 40 individuals, 21 expulsed the PIT tags and another 19 marked individuals were used for other behavioural experiments. Four antennas with length 4 m and height 0.4 m were situated in the pond approximately 5 m apart (Fig 3). If *T*. *cristatus* was moving in the longitudinal profile of the pond, it was guided to pass through the 4 m wide antennas using plastic barriers on the pond edges, which prevented movement of *T*. *cristatus* out of the tracking corridor (Fig 3). The antennas were periodically emitting a magnetic field that charges a PIT tag when present in the magnetic field. Subsequently, the charged PIT tag emits an individual code that is recorded and stored together with the date and time in the HDX Reader memory. The reader recording frequency was set to 10 energize and receive cycles s^-1^.

Since the experiment with releasing *T*. *cristatus* was started at the end of the breeding season, telemetry data captured the switch from aquatic to terrestrial phase. In a 72 h period we also tested differences of movement activity between males and females during day (5:00–21:00) and night (21:00–5:00). For each hour, we subtracted the number of detected females from the number of detected males and applied one sample t-test on these values. Prior to the analysis, we visually checked data for normality. Day and night settings were based on meteorological data from the Czech Hydrometeorological Institute. We also evaluated the differences in frequency of detections between males and females using the binominal general linear model (GLM) between males and females in their day/night activity. To do so, we compared detections (yes/no) of all newts between night and the following day. We had three comparable day-night sets in total, for each we tested the differences between males and females using the generalized linear model (GLM) assuming a binomial error structure, all in the statistical software R, version 3.4.3. [39].

## Results

The majority of *T*. *cristatus* survived the anaesthetics and surgery (98.57%) without any apparent bodily injuries (necrosis). Only two *T*. *cristatus* (one male and one female) died without euthanasia. They died without apparent cause of death (evaluated by the veterinarian) 40 hours after surgery from which they recovered well. After recovery from the surgery, all *T*. *cristatus* ate normally; movement and swimming behaviour was congruent with their behaviour before surgery without pathological signs (with only one exception).

**Fig 4:**
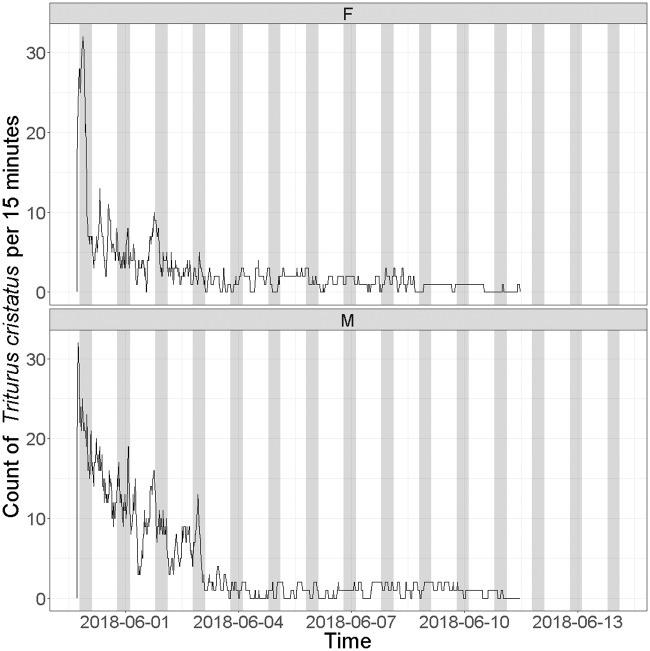
Detection rates. Detection rates in *T*. *cristatus* tracked by the passive telemetry system in the breeding pond. Upper panel shows counts of female newts and lower panel counts of male newts. White and grey stripes represent day and night periods.

**Fig 5:**
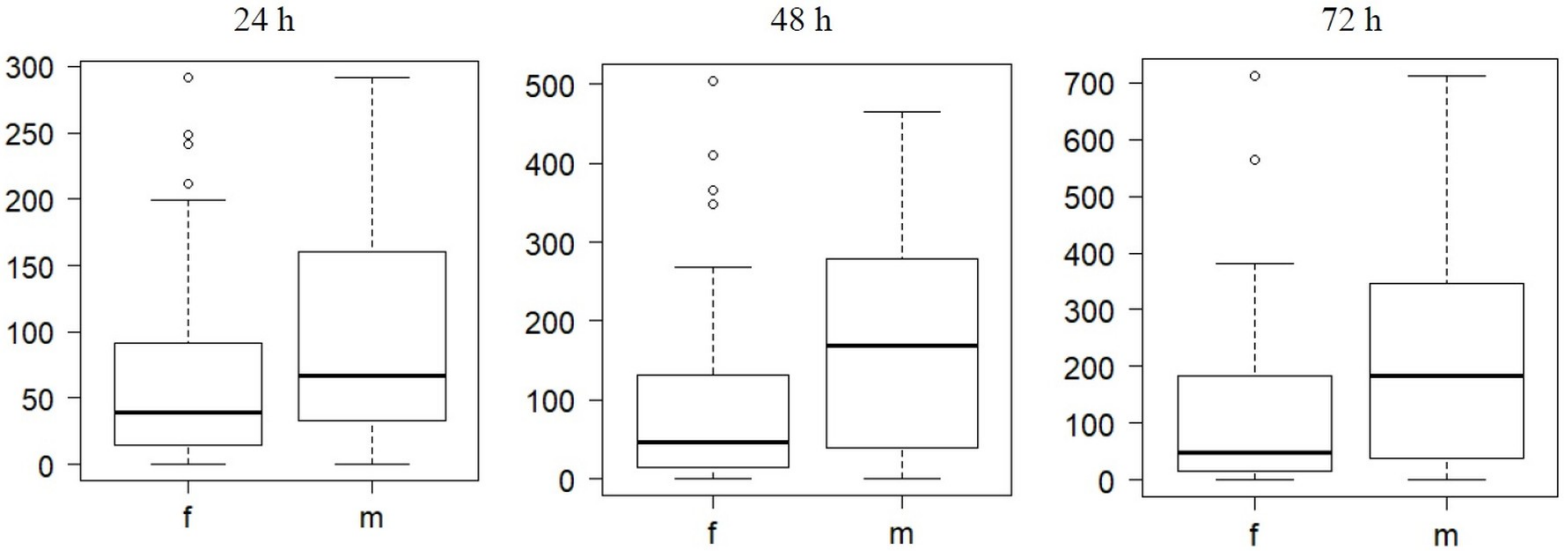
72 h experiment. Differences in detection during 72 h experiment, males (m) had significantly higher detection (F = 9.4, df = 1, P = 0.003) than females (f).

**Fig 6:**
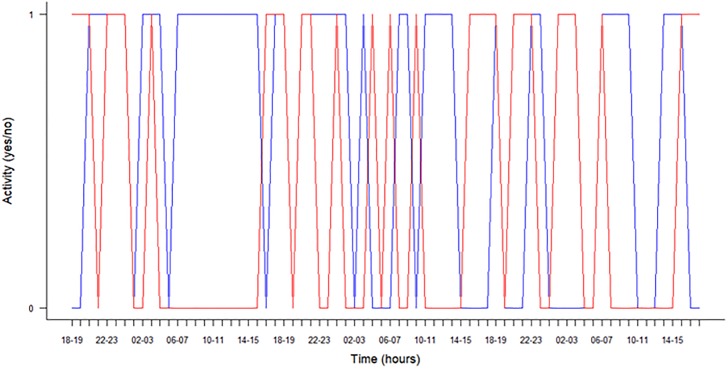
Diurnal activity. Diurnal activity during 72 h observation of the two most-detected individuals (Blue = male, red = female). Activity 1 (yes) means detection in one of the four antennas, 0 (no) means without detection.

When verifying the presence of PIT tag in the tagged *T*. *cristatus*, we discovered that 9 males and 12 females of all 140 PIT tagged individuals had been able to dislodge their PIT tags within three days after surgery. From the 100 PIT tagged *T*. *cristatus* released in the wild, we detected 97 individuals in the aquatic habitat by using four antennas connected with HDX Reader. The maximum number of recorded *T*. *cristatus* was on the date of release and we still registered a few individuals over the next next9 days. Altogether 24 106 records were obtained. Higher numbers of counts (detection rates) were recorded during the first 3 days of the experiment (Fig 4). In first 24 h, we detected 47 males and 49 females, in 48 h we had 32 detections of males and 16 of females, in 72 h we recorded 26 males and 12 females. Males had a significantly higher detection rate (F = 9.4, df = 1, P = 0.003, R2 = 8%) than females (Fig 5). On average, we captured 6.5 (95% confidence interval = 5.6 to 7.4, using t test R function, which assumes normal distribution) more males than females each hour (t = 14.6, df = 71, P <0.001). In two individuals that were more frequently detected (one female with 714 records and one male with 712 records), it was possible to create a graph of their diurnal activity during the 72 h after release (Fig 6) There was no significant difference in detection for males and females between day and night: first day (Dev = 0.32, df = 198, P = 0.57; binominal GLM), second day (Dev = 0.55, df = 198, P = 0.46; binominal GLM), third day (Dev = 1.59, df = 198, P = 0.21; binominal GLM). Other days were not tested, because lower detection rates.

## Discussion

Passive telemetry can bring wealth of information about life history of species under scrutiny. Depending on the study design, research can focus on diurnal activity or differences in movement between males and females. Another possibility is to tag individuals year prior to telemetry study and quantify arrivals and departures on the reproductive ground. Compared to the non-invasive photo-matching method, the PIT tag method requires catching the animal only once, with no further manipulation required [13]. The limitations of this method could be the high price of the reader or antenna and the reading range of the antenna [40, 41, 42]. The advantage of the HDX system is the ability to build an antenna of large size (up to 100 m), in comparison with the limited size of full duplex systems (FDX) antennas. In addition, FDX systems are more expensive, and in case of installation in flowing waters, they have to be fixed to prevent vibrations [19].

There are serious concerns about the possible negative impact of PIT tag surgery on species’ behaviour and growth [43, 44, 45]. Several studies investigated the possible negative effects on the body condition of different species of newts, or which compare PITs versus colouration patterns or other marking techniques [46, 47]. No significant effects of PIT-tagging on body condition and recapture rates were detected in long-term studies on *Triturus dobrogicus* and *Pelobates fuscus* by comparison with belly pattern recognition [46]. Because of high accuracy with regard to mark misreading risk, PIT-tagging appears to be the best available marking technique in amphibians, provided that the body size is sufficiently large for sheltering the transponder [48]. An implantable tag seems to be the best method for radio-tagging small and medium sized urodeles species such as *T*. *cristatus* [49] and subsequent tracking with passive telemetry.

For mark-recapture studies aiming at assessing dispersal, migration or survival, information regarding tag retention and failure rates is important for interpretation of results. PIT tag retention rates have been shown to vary among species, and even within species, depending on life stage, sex or location of tag placement [50, 51]. The position of the transmitters in the animal body is an important factor in determining the success of the study [46]. Methods using external tag attachment by elastic harness system or sutured to the skin seem unsuitable for telemetry studies of *T*. *cristatus*, because they could be easily lost [49]. On the other hand, telemetry studies with the tag positioned inside the body have previously been conducted on several species; however, most of them were larger than our model for amphibian species, *T*. *cristatus*–e.g. *Ambystoma maculatum* [52, 53], *A*. *tigrinum* [54] and *Desmognathus quadramaculatus* [55]. However, there might be several problems, e.g. with using the holding pen. In a study on the Bluehead sucker (*Catostomus discobolus*) one bluehead sucker died in the holding pen [56]. This fish was dissected and neither sign of internal damage nor puncture of internal organs was evident. The fish did have a lesion near its anal opening and a fungal infection which covered the anal opening and anal fin. The fungal infection in combination with the stress of being held in the holding pen may have contributed to its death.

The first aim of this study was to determine the survival rate of *T*. *cristatus* individuals carrying PIT tags for 48 h in the lab. In our study we implanted PIT tags under the skin of 140 *T*. *cristatus*; the survival rate of tagged *T*. *cristatus* was 98.57%. Such low mortality is common in PIT tagging studies, since it seems implantation of PIT tags into animal bodies does not usually cause high mortality [26]. Handling may indirectly lead to mortality [57], stress in e.g. fishes has been shown to have cumulative negative effects, including reduced growth and condition [58, 59]. Therefore, it is possible that in our study, the two dead individuals may be the result of a combination of several factors and not caused solely by surgery. If we compared it with an active telemetry, 87.5% of animals survived up to 48 days in the wild for successful radio-tracking surveys [60] post-surgery, until the battery life expired. By our results PIT tag implantation seemed to not impact the behaviour of *T*. *cristatus*, as already observed in other amphibian species [11, 60, 61, 62, 63, 64].

The survival rate can be also influenced mainly by the size and weight of the PIT tag [65]. Active (transmitters) and passive (PIT) tags differ in size due to presence or absence of battery, therefore PIT tags may be used for smaller organisms that transmitters. We calculated that the mass of the tag was around the 1.5% of the body mass of the *T*. *cristatus*, which is well within the recommended limits; for example study for active telemetry recommended a 10% limit [66]. In our study, the smallest (95 mm total length) male *T*. *cristatus* after 24h started to turn sideways, biting its skin; hence we decided to remove the PIT tag, euthanasia was not needed. Other newts (99.29%) were bigger (on average 102 mm) and the surgery did not affect their behaviour. In one study, the surgically implanted tags [67] in *T*. *cristatus* and *T*. *marmoratus* were 7.0–14.3% of the body mass. In another, the tags were 3% of the body mass of *T*. *cristatus* [49] in that study the authors also suggested that surgically implanted tags in *T*. *cristatus* may be up to 5–7% of the body mass without compromising neither animal welfare issues, nor the need for unbiased behavioural data. Although our sample size is small to make a conclusion about minimal body length, a 12 mm PIT tag may become a problem especially for small newts with standard length (tip of snout to posterior margin of cloacal lips) under 50 mm. The miniaturization process of PIT-tags and similar marking techniques has improved in recent years, being less invasive, and the size should continue to decrease in the future [26, 68].

The second aim of the study was to answer whether some PIT tags can be expelled. We observed a higher PIT tag expulsion rate value (15.71%). However, in a study of using PIT tags in *T*. *dobrogicus*, one-tenth of the individuals lost their tags [46]. One of the causes for such a high expulsion rate may be an overly large incision; the glass cover of the PIT tag is very smooth and it allows a sliding movement of the tags when incisions are not yet healed. The loss of the tags happened within the first week after surgery, before the X-ray was taken. This occurrence leads us to the consideration that future incisions could be sutured. Another option is to use a medical adhesive [11] or to insert the PIT tag more deeply into the body cavity [46]. However, amphibians have great regenerative abilities; a few days later only a scar is seen, therefore we decided to omit this procedure. Moreover, in fish, it was observed that sutures lead actually to an unnecessary increase in mortality [69]. This was the same in a study [11] where the newts had ruptured their sutures due to the solidified surgical adhesive. PIT tag expulsion has been described in other species, like fish, birds and rodents [50,70]. Results from the studies of long- and short-term retention rates and experiences from other types of surgically implanted transmitters [71] have led to the assumption that tag loss mainly occurs shortly after tagging for juvenile fish and during spawning for adults. That experiment started with 2 986 fish, and during the entire period PIT tags were not detected in 191 fish. Observed tag retention rates of 12.5 mm PIT tags implanted in the body cavity of Atlantic salmon over a 533 day period was 91% [72]. Low PIT tag retention rates of larger fish in studies of other Salmonidae species have been ascribed to tag loss during spawning [51, 73, 74]. In addition to tag expulsion with eggs, there are several ways tags can exit the fish: through the incision, through the body wall, and through the intestine [71]. Visual inspection of fish without tags from the fourth and fifth scanning did not reveal any obvious signs of tags having exited through the body wall [72]. In a study on birds, the overall PIT tag retention was 77.2% (N = 102); 12% (N = 6) of male and 29% (N = 24) of female tags were lost during the study period. Successful PIT tag retention depends on implanter’s experience and the attachment method [70]. In their study, once the implanter’s experience improved, retention increased from 69% (in the first five trapping sessions) to 88% (the last five trapping sessions). The percentage retention in study [70] of birds (~77%) was higher than in previous studies in non-passerines species: 30% for Adélie Penguins (*Pygoscelis adeliae*) [75] and 59% for Common Terns (*Sterna hirundo*) [76]. The loss rates of PIT tags vary, depending on the species of rodents, from 3.6 to 7.2% [77].

Finally we tested the reliability and possibility of using passive telemetry by detection of *T*. *cristatus* with PIT tags in a natural breeding pond. When we focused on practical use in the field by using four antennas in the breeding pond, we detected 97% of individuals in natural conditions. This is a similar number as in other studies [9, 17].*T*. *cristatus* are predominantly nocturnal [29, 78, 79]. Males may show additional daytime activity during the peak of the breeding season [29]. Our results of activity showed that major detections were between 18:00–24:00 and lower records between 10:00–14:00, however, when we look at individuals, there is great variability and clearly there is no hour without detection (same for males and females). Generally, both sexes showed great variability in their activity. In our experiment, we observed that males had a higher detection rate than females. The result of movement behaviour showed that e.g. male of *T*. *cristatus* in a breeding pond can travel up to 20 m between antennas in 78 seconds (see data set).

Ease and safety of application of anaesthetics are additional criteria [36]. Isoflurane is considered effective, but it is expensive, difficult to apply (particularly in the field) and its vapours are toxic for the investigator [80]. Application of other anaesthetics by injection may not be possible in subjects that are difficult to capture and hold without damage (e.g., salamanders). Phenolic compounds, such as eugenol, an active component of clove oil, is an effective anaesthetic for treating amphibians that can be applied by immersion, potentially making it suitable for work in the field with species that are difficult to handle [81]. However, in a study on African clawed frogs (*Xenopus laevis*), it has been documented that the duration of clove oil anaesthesia lasted longer in larger bodied individuals [38]. In our study, we did not observe any problems with the anaesthetic. The duration of the surgical depth of anaesthesia (60–90 minutes) gives plenty of time for surgical procedures. It is necessary to keep the skin moist during handling and the recovery time, as dermal respiration is assumed to be sufficient to oxygenate the circulatory system and prevent clinical hypoxemia [81]. The newts can be removed from the anaesthetic bath before the cessation of gular respiration, because there is a time lag of two–three minutes between induction of anaesthesia, i.e. loss of righting reflex and abdominal respiration, and subsequent apnoea [50]. This could reduce the recovery period, which offers an important recommendation for field studies.

To sum up, our study reports the partially successful use using of PIT tags in the body of *T*. *cristatus*, as a small amphibian species using clove oil as anaesthetic. Although we report small mortality and substantial PIT tag loss, we conclude that this promising method could facilitate the detection of recaptured individuals in long-term studies. We cannot exclude that there may be later expulsion of PIT tags; however, thanks to the double mark approach the possibility of identification of individuals can be made on the comparison of photos in a database, provided that the individual occurs at the same site. If the number of lost tags is stable for a certain period and can be quantified, it can be accounted for, and thus the mark-recapture or telemetry study does not have to be unsuccessful. PIT tag usage allows the observation of marked small animals of different species in aquatic as well as in terrestrial habitats, providing data on their dispersal, diurnal activity, movement behaviour and generally behavioural patterns. This promising method had no significant effect on welfare of newts, no signs of stress and pathological changes in behaviour were observed. However, it is important to assess estimates of PIT tagging mortality and PIT tag loss before making population estimates based on acquired data. Moreover, it is necessary to note that our data were affected by the short time of observation, because the breeding pond completely dried up.

## Supporting information

S1 Table Supporting dataset. Field survey data.
(XLS)
